# Supplementary material for: Quantifying confidence shifts in a BERT-based question answering system evaluated on perturbed instances
Source: PLoS One. 2023 Dec 20;18(12):e0295925. doi: 10.1371/journal.pone.0295925 (PMC10732378; doi:10.1371/journal.pone.0295925)
Supplement: Attachment — Submitted filename: revision-letter.pdf [file pone.0295925.s001.pdf]

We thank the editors and all the reviewers for their comments and have attempted to take each concern into account. Among the most significant changes to the revised paper is a more detailed Related Work and Discussion section, and fewer subheadings in other sections. Below, we provide a point-by-point response.

Thank you for stating the following in the Acknowledgments Section of your manuscript:
"This work was funded as part of MOWGLI, a project in the DARPA Machine Common Sense (MCS) program, supported by the United States Office Of Naval Research under Contract No. N660011924033."
We note that you have provided funding information that is currently declared in your Funding Statement. However, funding information should not appear in the Acknowledgments section or other areas of your manuscript. We will only publish funding information present in the Funding Statement section of the online submission form.
Please remove any funding-related text from the manuscript and let us know how you would like to update your Funding Statement. Currently, your Funding Statement reads as follows:
"MK received funding for this work as a principal investigator of MOWGLI, a project in the Defense Advanced Research Projects Agency (DARPA) Machine Common Sense program, supported by the United States Office Of Naval Research under Contract No. N660011924033. The funders had no role in study design, data collection and analysis, decision to publish, or preparation of the manuscript."
Please include your amended statements within your cover letter; we will change the online submission form on your behalf.

<u>Response:</u>
We have removed the funding information from the manuscript and the current funding statement looks fine to us.

<u>Reviewer #1: This paper evaluates the confidence shifts in a ROBERTa model on perturbing QA instances.</u>

#Introduction
In the introduction, the authors explained BERT-like systems and described their choice of ROBERTa. I suggest a paragraph arguing in favor of ROBERTa instead of a "highly optimized version".

<u>Response:</u>
We have revised the introduction and related work section to offer a more comprehensive

explanation of the specific advantages of RoBERTa, highlighting how its modified training techniques and superior performance on NLP benchmarks make it a suitable choice for our research.

They argue that they provide a new set of three confusion probes to test linguistic properties. I suggest removing "linguistic properties" and explaining the three probes later, as the authors did.

Response:
We have removed “linguistic properties” and rephrased the original sentence “We design and implement a novel set of three confusion probes to test linguistically relevant properties ” to “We design and implement a novel set of three confusion probes to observe how confidence distribution shifts”.

# Related Work
Concerning their related work, it would be interesting to have a ROBERTa discussion in a BERT-based layer of linguistic approaches, the main differences, for example.

Response:
We have rephrased the RoBERTa paragraph in Related Work section to provide a more detailed explanation of the main differences between RoBERTa and BERT. We believe that this addition enhances the manuscript by offering readers a clearer understanding of the nuanced differences between these two influential models.

Some typos
on on -> on
C(A,P) -> C(Ã,P)

Response:
We appreciate your careful review of our manuscript and have corrected the mentioned typos.

Reviewer #2: This research investigates a BERT-based question-answering model's response to confusion probes, revealing that despite perturbations, the model often maintains a preference for its original correct answer while exhibiting random confidence distributions for incorrect answers, suggesting potential avenues for further analysis and highlighting the need for additional research into transformer-based model behavior and biases.I find the research

questions about what might happen if no correct answer exists for a given prompt among the provided set of choices quite interesting. Moreover, the manuscript is well-written. Motivation and methodology are sound. Therefore, my overall opinion is positive. I have just some comments to help improve the manuscript:

- The paper focuses exclusively on BERT. I missed a discussion about whether the results are generalizable to other types of models of the same nature.

Response:
In the revised manuscript, we introduced a new section within the Discussion Section, featuring supplementary experiments employing the GPT-3.5 Turbo model. Given the widespread popularity and exceptional performance of GPT models, we posit that these additional experiments offer robust and representative evidence to support the generalizability of our findings. The results show that while the specific proportions of shifts may exhibit some variance, the general trends in confidence shifts remain remarkably consistent across different models.

- I do not know if the authors intend to be exhaustive in all the domains in which BERT has application, but some very important ones need to be included, for example, LegalBERT.

Response:
Thanks for the valuable suggestion. We have included more domain-specific applications in the related work section of the revised paper. This includes mention of LegalBERT, ClinicalBERT, FinBERT, and PatentBERT, among others. We think the current Related Work section can provide a more comprehensive overview of the significance of the BERT model in various fields.

- Very little information is given about the RoBERTa model chosen for the experiments. This brings two additional problems: a) it is necessary to go to the literature to understand the details about the training of such a model, and b) it needs to be clarified why such a configuration has been chosen over many other existing ones.

Response:
We have expanded upon the pre-training and fine-tuning details of RoBERTa in both the Related Work and Materials and Methods sections in the manuscript, aiming to offer readers a clearer understanding of our model choice and setup.

Additionally, regarding the selection of specific fine-tuning parameters, our choice was based on optimizing the model's performance on the validation set. We selected the configuration that yielded the best accuracy results during the fine-tuning process. We hope that these clarifications provide more insight into our model selection and fine-tuning decisions.

- *The following comment is closely related to the previous one. Can the authors foresee analogous behavior/results if the model configuration is changed?*

<u>Response:</u>
We appreciate your comment regarding the potential impact of altering the model configuration on our results. While we did not replicate the experiments by varying the RoBERTa model configuration, we did conduct experiments across different models, including ChatGPT (now presented in the Discussion Section), UnifiedQA, and XLNet. Across these diverse models, we observed a certain degree of consistency in the confidence shift patterns, which suggests that the observed behavior is not solely dependent on the specific architecture but is a characteristic of transformer-based language models in general.

Given this observation, we believe that even if we were to modify the configuration of the RoBERTa model, it is likely to exhibit similar behavior in terms of confidence shifts when exposed to perturbations. However, further experimentation would be needed to confirm the extent of this similarity and explore the nuances of different configurations.

- *I do not fully understand the sentence: "incorrect choice that appears superficially closest to the prompt." Does it mean the choice with a smaller semantic distance to the prompt is chosen? How is this defined?*

<u>Response:</u>

The phrase 'superficially closest to' does indeed relate to a smaller semantic distance, but it primarily emphasizes a more 'superficial' characteristic, such as the presence of common words shared between the choice and the prompt. To enhance clarity, we have rephrased the original sentence, and the current version reads:

'In the case of choice-based perturbation, the model will choose the incorrect choice that, on the surface, seems most closely aligned with the prompt, typically by sharing a greater number of common words, despite its incorrectness.'
